# A Blockchain Technology Introduction Strategy for Asymmetric Sharing Platforms under Different Homing Behaviors of Both Sides

**DOI:** 10.3390/ijerph192316060

**Published:** 2022-11-30

**Authors:** Libin Guo, Xiangtian Guo

**Affiliations:** School of Management Science, Qufu Normal University, Rizhao 276826, China

**Keywords:** sharing economy, asymmetric platforms, blockchain technology, multi-homing behavior, privacy concerns

## Abstract

To address user privacy concerns and improve user trust levels, sharing platforms are commencing to focus on investing in blockchain technology. This study focuses on blockchain technology investment and pricing strategies for two asymmetric sharing platforms. By constructing a Hotelling model, we investigate the investment strategies of the two asymmetric platforms regarding blockchain technology under different user attribution behaviours, i.e., single-homing or multi-homing, and the optimal pricing under different investment decisions. Afterwards, we compare and analyse the investment strategies under different conditions, obtain the influence of relevant market factors on the pricing strategies of the platforms, and finally determine the optimal timing of blockchain technology investment for asymmetric sharing platforms. The results indicate that when users’ perception of blockchain value is high, both platforms are motivated to introduce blockchain technology, and, conversely, a stronger platform exits the blockchain market. In multi-homing markets, platforms are more likely to implement blockchain strategies and the cost of technology investment is significantly higher than in a single-homing market. In addition, we also find that the degree of differentiation has a significant impact on the blockchain strategies of weaker platform under multi-homing market.

## 1. Introduction

For sharing economy platform, such as Airbnb and Uber, the trust of users is the critical factor for its success [1,2,3,4]. The importance of this can be attributed to the simple reason that consumers will only participate in platform trading services if they have a certain level of trust in the platform [5,6,7]. However, sharing economy platforms confront a challenge in enhancing user trust: privacy concerns [3,8,9,10]. As exemplified by YouTube’s 2016 data breach, the personal information of some 2.7 million of its UK users was stolen by hackers. Not coincidentally, the Facebook-Cambridge Analytica user privacy data breach put users at risk by improperly protecting their sensitive personal information and invading their privacy, which ultimately resulted in consumer sensitivity to privacy concerns being at a record high [7]. It is clear that the sharing economy of peer-to-peer transactions has increased information risks, and the privacy concerns of individuals are consequently made more serious and complex. Therefore, it is essential to explore trust and privacy together.

Justice theory has been broadly applied recently to address those factors which can have an immediate negative impact on personal privacy concerns [11]. The presence of justice demonstrates platform’s focus on privacy concerns providing users confidence and control [12]. Therefore, platforms can mitigate consumer privacy concerns by providing a fair and trustworthy environment [11,12]. Many experts believe that blockchain is one of the most disruptive technological innovations of recent times and has emerged as a new approach to solving problems related to recording, tracking, verifying and aggregating various types of information [13,14]. Considering the features of blockchain technology, Scholars argue that a sharing economy based on blockchain technology can facilitate peer-to-peer transactions [15,16,17]. For example, Hawlitschek et al. [18] and Tan and Salo [17] advocate that blockchain technology should be nested in a sharing platform and serve a decentralized function in the sharing economy. It is clear that blockchain technology will transform the future of digital transactions in the sharing economy and change the nature of digital trust. Furthermore, digital trust in blockchain reflects the level of consumer trust and helps users make rational judgments between security and privacy [19]. However, since the blockchain-based sharing economy is an emerging technological phenomenon, little is known about whether participants in the sharing economy believe that the fair practices used by platforms to address procedural justice are effective in protecting their privacy and thus will have greater trust in sharing economy platforms. This research investigates the degree of value perceived by different types of users of blockchain technology and its impact on behavioral intention by highlighting privacy concerns and how trust affects users’ behavioral intention in the sharing economy, thus identifying the optimal timing for the introduction of blockchain technology for competing sharing economy platforms.

Additionally, as a typical bilateral platform, the sharing platform can further expand its user scale through the cross-edge network effect of users. The higher the number of users on one side, the higher the utility of the users on the other side [20]. However, multi-homing behavior changes user utility and scale, complicating platform pricing and investment decisions. For example, in the case of car sharing, a driver may drive for both Uber and Lyft, and a passenger may use the apps of both companies [21]. Therefore, it is necessary to take into account the multi-homing behavior of users in the investigation of the above problem.

Motivated by the above practices, we attempt to answer the following research questions in this paper: (1) How does the level of user trust in blockchain affect the timing of blockchain technology introduction in asymmetric competitive sharing platforms? (2) In such a market environment, what impact does multi-homing behavior have on platforms’ decisions compared to users’ single-homing behavior? (3) How market factors such as the differentiation of sharing platforms and the level of consumer trust affect platform pricing under different consumer homing behaviors? (4) Considering different market conditions, do asymmetric sharing economy platforms have the opportunity to achieve blockchain equilibrium in competition?

To address these problems, we consider a duopoly characterized by platform competition, where two sharing platforms receive service fees by matching supply and demand. The platforms are asymmetric with different independent intrinsic values. Users choose which sharing economy platform to trade their services based on service preferences and other horizontal differentiation factors. Ultimately, platforms determine investment and pricing strategies based on market demand for their services. Specifically, in a bilateral user single-homing scenario, we study the investment decisions of two asymmetric platforms regarding blockchain technology, and the optimal pricing under different investment decisions. Then, we extend to the optimal decision of platforms when bilateral users are multi-homing. After that, we compare and analyze the investment strategies of the platforms to obtain the impact of different market factors on the pricing strategies of the platforms, and finally determine the optimal blockchain technology investment timing for asymmetric sharing platforms.

The remaining parts of this paper are organized as follows. Section 2 reviews the relevant literature. Section 3 describes the game model in different contexts. Section 4 analyzes the equilibrium results in different contexts. Section 5 derives the equilibrium strategy by comparing and analyzing the optimal strategy. Finally, the above conclusions are summarized in Section 6.

## 2. Literature Review

There are three closely related streams of literature: (1) research on privacy concerns in the sharing economy; (2) research on trust in blockchain technology, and (3) research on the behaviours of single-homing and multi-homing.

### 2.1. Privacy Concerns in the Sharing Economy

The first research stream has further fueled research interest in the sharing economy over recent years. Privacy concerns indicate skepticism and caution about online services [9], exist in different online contexts, and potentially influence the consumption behavior [22]. Early research on online privacy concerns mainly centered on the specific contexts of e-commerce service [23]. The emergence of the sharing economy has brought privacy concerns to a new level [4]. The exchange process that occurs in the sharing economy requires comprehensive personal information to be shared by consumers on and off the platform [10], which will cause users to be vulnerable to losing control over the dissemination and usage of this information [24]. The more prominent users’ privacy concerns are, the less probably they are to engage in online transactions [23]. Zarifis et al. [3] explore how to reduce privacy concerns in the sharing economy and indicate that language has a role in interaction, but this role is limited, while platform norms and habits have a greater impact in addressing privacy concerns. However, in the context of the sharing economy, concerns about online and physical privacy are intertwined, neither can be avoided if users decide to participate in sharing [9]. Although online privacy concerns may be high in general, users may choose to interact with specific institutions that they judge to be trustworthy [24,25]. Lutz et al. [9] investigate the role of privacy concerns in the sharing economy and confirm that users seem quite content to share goods and services, despite the privacy concerns associated with such sharing. Furthermore, Lutz et al. [9] point out that the privacy guarantee will alleviate users’ online privacy concerns and enhance their trust in the platform. Therefore, platforms can alleviate consumers’ privacy concerns by providing a fair and trustworthy context [11,26]. In this study, we will focus on the privacy interventions in the context of the increasingly growing sharing economy. Procedural justice is shaped by granting consumers control over information disclosure and privacy protection actions, which has a positive impact on mitigating privacy concerns [4,8]. We hypothesize the effect of a fair information practice specifically targeted at procedural justice, that is, the blockchain technology.

### 2.2. Trust in Blockchain Technology

The literature which concerns digital trust in blockchain are less saturated. Digital trust in blockchain can be described as a rational judgment made by users between security and privacy, capturing their level of confidence [19]. In fact, most of the blockchain literature is often discussed in the context of trust for which it is described as trustworthy, implying that blockchain technology can be trusted by virtue of its design [27,28]. For instance, Wang et al. [29] analyze the motivation of platforms to implement blockchain technology to verify their disclosures and thus eliminate consumer mistrust. Lumineau et al. [30] argue that blockchain technology is a governance mechanism because it helps to build credible reputation systems and achieve transparency among different organizations in the sharing economy. Fiorentino and Bartolucci [31] propose adopting blockchain-based management system as new governance techniques to advance the traceability, transparency and decentralization of transaction in the sharing economy. However, as the blockchain matures, the level of trust users have in the services, interactions and organizations behind it becomes increasingly critical [32]. Security and privacy are essential to blockchain technology, as it permits its existence free of authorized third parties. Shin [19] explore the impact of users’ perceptions of security on platforms’ intentions to adopt blockchain, and confirm that consumer attitudes towards blockchain have a positive impact on the intention of adopting blockchain. On an intermediary platform, security may depend on more than just the technical aspects of security [33]. Trust is a critical component in the decision to adopt blockchain technology [34]. Zavolokina et al. [35] investigate how to implement design elements that support trust to promote user trust in blockchain platforms. While many researches have investigated the impact of perceived security in different contexts, only a few have applied it to the blockchain context. In the case of blockchain, users may not assess security at the same level as physical security [36]. While the assessment of technology is based on scientific solutions, it is the individual’s level of assessment that influences intentions and behavior [37]. In accordance with ongoing research, our study investigates the impetus and timing of introducing blockchain technology to platforms from a user-centric perspective.

### 2.3. The Behaviours of Single-Homing and Multi-Homing

Another relevant research focus is on consumers’ homing behavior. Consumers’ homing behavior can generally be categorized into into two types: single-homing and multi-homing. Single-homing refers to users using only one platform [38], that is, users are re-constrained to a single platform [39]. Multi-homing refers to users using multiple platforms [38], that is, users access services provided by two competing platforms [40]. The majority of the existing literature on multi-homing is limited to one side of the market [39,41,42]. For example, Belleflamme and Peitz [39] point out that if one side is multi-homing, the platforms compete on the single-homing side and exert monopoly power on the multi-homing side. Ambrus et al. [43] examine the impact of strategic interactions between media on advertising levels, as well as the impact of entry and consolidation, within the context of consumer multi-homing. Additionally, Armstrong and Wright [44] reveal that equilibrium does not exist in the presence of multi-homing on both sides of the market, whereas equilibrium with multi-homing occurs only on one side. However, our model considers the case where both supply and demand bilateral users are multi-homing, and demonstrates that the multi-homing phenomenon can exist in equilibrium in both sides. As Bakos and Halaburda [45], Li and Zhu [46] indicated, this equilibrium is the most common distribution in reality.

Specifically, Table 1 presents a summary of the available literature and novelty of this paper.

## 3. Model

We consider a duopoly characterized by platform competition where both platforms (*A*, *B*) developed a shared economic platform connecting supply and demand sides such that they acquire platform service fee from matching supply with demand. Each platform is different in terms of service quality and service efficiency, thus bringing different stand-alone (intrinsic) values to users. Users choose which shared economic platform to make service transactions based on service preferences and other horizontal differentiation factors. We abstract the distribution of these taste preferences on both the supply (superscript *I*) and demand side (superscript II) through a Hotelling line which makes platforms *A* and *B* are horizontally differentiated and are located on the extremes. In which, the brand independent value of platform *A* is higher than that of platform *B*. We use ψ to describe the degree of brand differentiation, which $\psi =\frac{A}{B}>1$. The lager the ψ, the greater the brand differentiation. The supply and demand side are spatially differentiated and uniformly distributed on their service preferences. As with the existing location model, each point represents an ideal service trading platform for both the demand side and the supply side. And they suffer from negative utility (tx or ty) when obtaining services located at a distance x(y) from their ideal trading platform. In our model, the parameter t(t>0) can be described as the degree of differentiation, the greater the *t* the greater the differentiation. In other words, the parameter *t* also captures the loss of regret due to platform differences, with larger values indicating a greater degree of regret. Therefore consumers are more adversely affected when being forced to choose a platform which they do not like to trade services on.

We improved our model along the lines of Armstrong and Wright [44], and both sides of the shared economic platform produced a network external cross effect, that is, revenue from the supply side depends on the number of participants in the demand side and the demand side also gain the value from the number of participants in the supply. Note that we assume that the cross-side externality coefficient is the same for the demand and supply-side and is given by α, where α abstracts the marginal value of adding a demand side to the supply side and adding a supply side to the demand side.

### 3.1. Platform Blockchain Strategies

The main purpose of this paper is to determine the timing of the introduction of block chain technology into the shared economic platform. We specifically model the mechanism through which platforms impact profitability with introducing blockchain technology. Blockchain technology is a database-based technology which stores and distributes data among all stakeholders involved in the network, and thus can ensure the reliability, traceability and authenticity of information on the Internet where trust cannot be established. In addition, some users are sensitive to block chain technology, that is, they have high requirements for personal privacy protection. When these segments are traded on platforms which use block chain technology, their utility is significantly reduced. There are also some sensitive users who have high requirements on the authenticity and traceability of the products. When these segments conduct service transactions on platforms which use block chain technology, their utility will be significantly improved. Therefore, the platform needs to consider the composition of the market when deciding whether to introduce blockchain technology to enhance the competitiveness of the platform.

#### 3.1.1. Introducing Blockchain Technology

We define this strategy as one where the platform chooses to introduce blockchain technology with a one-off fee *f*. In this case, a proportion of users, represented by η (0<η<1), is blockchain technology-sensitive. These are denoted as type η users, and represented by subscript η. This ratio η is exogenous and related to users’ educational background and consumption habits. A type η users obtain from joining the respective platform are $\theta v_{A}$ and $\theta v_{B}$. When the blockchain-sensitive user is a high-valuation type, θ>1. And When the blockchain-sensitive user is a low-valuation type, 0<θ<1. In addition, a type (1−η) users which is blockchain-insensitive obtain from joining the respective platform are $v_{A}$ and $v_{B}$, when the platforms introduce blochchain technology. Therefore, when characterizing the utility function, we need to consider two types of users: η and 1−η. All decisions under this strategy are denoted with a superscript *Y*.

#### 3.1.2. Without Introducing Blockchain Technology

We define this strategy as one where the platform chooses not to introduce blockchain technology and thus the platform does not incur any cost in technology introduction. As a result, users rely entirely on the platform’s independent intrinsic value to set their expectations. All decisions under this strategy are denoted with a superscript *N*.

### 3.2. Relevance of Strategy and Homing

The research on the timing of introducing blockchain into the platform will be incomplete without considering certain market conditions commonly observed in platform competition. Note that users will play an important role if the market conditions do not allow them to switch to the other platform when they select a platform. In such a market, the users may be considered as loyal agents. However, when the users multi-homing, they will ultimately affecting platforms’ decisions regarding the timing of blockchain technology introduction. Similarly, many authors [44,47] in the model defined by the Hotelling condition have examined some markets where users end up serving both sides of the market. For example on ridesharing platforms, a driver may join both Uber and Gift, while a passenger may place an order both on Uber and Gift. Without a rigorous analysis, it is not clear how the timing of the introduction of pre-blockchain technology is affected by such market conditions.

To examine the timing of the introduction of the platform blockchain technique under the multi-homed market conditions, we first develop the basic case that the users are single-homed, and when the platforms do not introduce the blockchain technology, their perception of the intrinsic value of the platform remains unchanged. We denote this with a superscript SN. Based on this basic case model, we developed our case model where the users are still single-homing, but now the platforms introduce blockchain technology, denoted with a superscript SY. In Section 4.2, we discussed multi-homing option and examine both cases where platforms do not introduces the blockchain technology (MN) and introduce blockchain technology (MY). Through the analysis of the above four situations, we can clearly separate the relationship between strategic choices and typical market conditions.

### 3.3. Timeline of the Model

**Figure 1:**
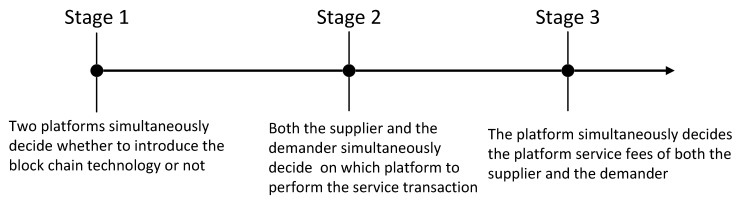
Timeline of the game.

In our model, we assume that the game is divided into three stages. In addition, users who are homogeneous in all aspects may still have different expectations of the same platform as a response to the strategic timing of the platform’ s block chain technology. Therefore, it is important to develop the expected utility for each strategy separately. Specifically, the timeline of the game is illustrated in Figure 1 and developed as follows:

Stage 1: Under the traditional strategy, neither platform introduces blockchain technology, and both of them provide services through traditional shared platforms. Thus the platform does not need to pay extra technology introduction cost, and the blockchain-sensitive user does not generate positive or negative perception emotions on the platform. Under the blockchain strategy, not only does the platform have to pay the one-time blockchain technology introduction fee, but it also needs to consider the different perceptions of the users towards blockchain when pricing. Based on the Hoteling model, we conceptualize the end product market as a straight line, and specify the external positions of platform *A* at 0 and platform *B* respectively, with a distance of 1 [44].

**Figure 2:**
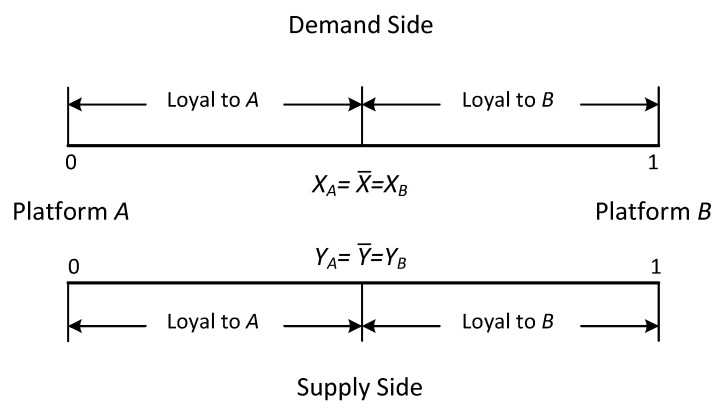
Single-homing.

**Figure 3:**
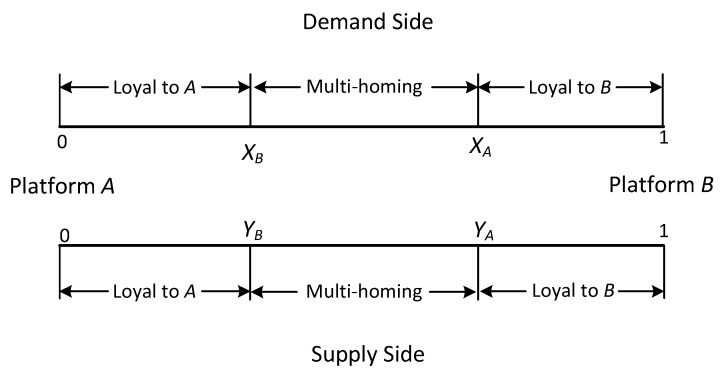
Multi-homing.

Stage 2: The supply and demand sides are uniformly distributed along lines representing the market, and their preferences vary according to their location. We assume that users can only transact once on the same platform. Specifically as shown in Figure 2 and Figure 3.

Stage 2.1.1: For the base case of single-homimg, a demander located at $x_{A}$ on side *X* (respectively a supplier located at $y_{A}$ on side *Y*) receives utility from joining platform i∈A,B under the traditional strategy:

(1) $$\begin{array}{cc} U_{A}^{I-SN} & =v_{A}+\alpha n_{A}^{II-SN}-p_{A}^{SN}-tx\\ U_{B}^{I-SN} & =v_{B}+\alpha n_{B}^{II-SN}-p_{B}^{SN}-t\left(1-x\right)\\ U_{A}^{II-SN} & =v_{A}+\alpha n_{A}^{I-SN}-w_{A}^{SN}-ty\\ U_{B}^{II-SN} & =v_{B}+\alpha n_{B}^{I-SN}-w_{B}^{SN}-t\left(1-y\right) \end{array}$$

where $v_{i}\left(i\in \left\{A,B\right\}\right)$ represents the intrinsic value gained through the platforms; $\alpha n_{i}^{J-SN}\left(i\in \left\{A,B\right\},\iota \in \left\{I,II\right\}\right)$ represents the cross-side externality utility of the other side; $p_{i}^{SN}\left(i\in \left\{A,B\right\}\right)$ represents platform services fees from the demand side, while $w_{i}^{SN}\left(i\in \left\{A,B\right\}\right)$ represents platform services fees from the supply side; and for platform *A* a crowd of $x_{A}$ demanders participate on side *X* and a crowd of $y_{A}$ suppliers participate on side *Y*, while for platform *B* a crowd of $1-x_{B}$ demanders participate on side *X* and a crowd of $1-y_{B}$ suppliers participate on side *Y* and parameter *t* represents the degree of differentiation between platforms.

Then we obtain the demand functions of platform *A* and platform *B* with respect to the supply side and the demand side under scenario SN:

(2) $$\begin{array}{c} n_{A}^{I-SN}=\frac{-\alpha^{2}+\alpha A-tp_{A}^{SN}-\alpha w_{A}^{SN}+At-\alpha B+tp_{B}^{SN}+\alpha w_{B}^{SN}-Bt+t^{2}}{2t^{2}-2\alpha^{2}}\\ n_{B}^{I-SN}=\frac{-\alpha^{2}-\alpha A+tp_{A}^{SN}+\alpha w_{A}^{SN}-At+\alpha B-tp_{B}^{SN}-\alpha w_{B}^{SN}+Bt+t^{2}}{2\left(t^{2}-\alpha^{2}\right)}\\ n_{A}^{II-SN}=\frac{-\alpha^{2}+\alpha A-\alpha p_{A}^{SN}-tw_{A}^{SN}+At-\alpha B+\alpha p_{B}^{SN}+tw_{B}^{SN}-Bt+t^{2}}{2t^{2}-2\alpha^{2}}\\ n_{B}^{II-SN}=\frac{-\alpha^{2}-\alpha A+\alpha p_{A}^{SN}+tw_{A}^{SN}-At+\alpha B-\alpha p_{B}^{SN}-tw_{B}^{SN}+Bt+t^{2}}{2\left(t^{2}-\alpha^{2}\right)} \end{array}$$

Stage 2.1.2: Under the blockchain strategy, for blockchain-sensitive users, they may generate a positive or negative perception of the blockchain, which will affect their expected utility, while for blockchain-insensitive users, their expected utility is not affected by the blockchain. And thus, for a type η blockchain-sensitive users, a demander located at $x_{A}$ on side *X* (respectively a supplier located at $y_{A}$ on side *Y*) receives utility from joining platform i∈A,B is as follows:

(3) $$\begin{array}{cc} U_{A}^{I-SY} & =\theta v_{A}+\alpha n_{A}^{II-SY}-p_{A}^{SY}-tx\\ U_{B}^{I-SY} & =\theta v_{B}+\alpha n_{B}^{II-SY}-p_{B}^{SY}-t\left(1-x\right)\\ U_{A}^{II-SY} & =\theta v_{A}+\alpha n_{A}^{I-SY}-w_{A}^{SY}-ty\\ U_{B}^{II-SY} & =\theta v_{B}+\alpha n_{B}^{I-SY}-w_{B}^{SY}-t\left(1-y\right) \end{array}$$

where θ>1 represents that users have a positive value perception of the platform where the blockchain technology is introduced, respectively, 0<θ<1 represents that users have a negative value perception of the platform where the blockchain technology is introduced. we assume the users with full coverage and single-homing on both sides. There is also a segment of undifferentiated users in the market, i.e., they acquire the same expected utility from both Platform *A* and Platform *B*. In this case, $x_{A}=\tilde{x}=x_{B}$ $\left(n_{A}^{I-SN}=\tilde{x}=n_{B}^{I-SN}\right)$, s.t. $U_{A}^{I-SN}=U_{B}^{I-SN}$ and similarly $y_{A}=\tilde{y}=y_{B}$ $\left(n_{A}^{II-SN}=\tilde{y}=n_{B}^{II-SN}\right)$, s.t. $U_{A}^{II-SN}=U_{B}^{II-SN}$.

Then we obtain the demand functions of platform *A* and platform *B* with respect to the supply side and the demand side under scenario SY

(4) $$\begin{array}{c} n_{A}^{I-SY}=\frac{\alpha \left(w_{B}^{SY}-w_{A}^{SY}\right)+\left(\alpha +t\right)\left(-\alpha +A\eta \left(\theta -1\right)+A+B\left(\eta \left(-\theta \right)+\eta -1\right)+t\right)-tp_{A}^{SY}+tp_{B}^{SY}}{2(t-\alpha )(\alpha +t)}\\ n_{B}^{I-SY}=\frac{\alpha \left(w_{A}^{SY}-w_{B}^{SY}\right)+\left(\alpha +t\right)\left(-\alpha +A\left(\eta \left(-\theta \right)+\eta -1\right)+B\eta \left(\theta -1\right)+B+t\right)+tp_{A}^{SY}-tp_{B}^{SY}}{2(t-\alpha )(\alpha +t)}\\ n_{A}^{II-SY}=\frac{t\left(w_{B}^{SY}-w_{A}^{SY}\right)+\left(\alpha +t\right)\left(-\alpha +A\eta \left(\theta -1\right)+A+B\left(\eta \left(-\theta \right)+\eta -1\right)+t\right)-\alpha p_{A}^{SY}+\alpha p_{B}^{SY}}{2(t-\alpha )(\alpha +t)}\\ n_{B}^{II-SY}=\frac{t\left(w_{A}^{SY}-w_{B}^{SY}\right)+\left(\alpha +t\right)\left(-\alpha +A\left(\eta \left(-\theta \right)+\eta -1\right)+B\eta \left(\theta -1\right)+B+t\right)+\alpha p_{A}^{SY}-\alpha p_{B}^{SY}}{2(t-\alpha )(\alpha +t)} \end{array}$$

Stage 2.2.1: We now allow multi-homing on the demand and supply sides of the platforms. Demand-side market coverage of platform *A* is given by $x_{A}$, and the platform *B* is given by $1-x_{B}$. Multi-homing on the demand side occurs when $x_{A}>x_{B}$. Respectively, supply-side market coverage of platform *A* is given by $y_{A}$, and the platform *B* is given by $1-y_{B}$. Multi-homing on the demand side occurs when $y_{A}>y_{B}$. Specifically as shown in Figure 3. Note that the utility of multi-homing users is equal to the sum of the utility of joining each platform. In our model, we assume that there is no double counting of cross-externality benefit, while double counting of the instrinsic benefit of the two platforms.

Thus, under the traditional strategy, a multi-homing demander located at $x_{A}$ on the side *X* (respectively a multi-homing supplier located at $y_{A}$ on the side *Y*) receives utility from joining platforms:

(5) $$\begin{array}{cc} U_{A\&B}^{I-MN} & =A+B+\alpha -p_{A}^{MN}-p_{B}^{MN}-t\\ U_{A\&B}^{II-MN} & =A+B+\alpha -w_{A}^{MN}-w_{B}^{MN}-t \end{array}$$

Then we obtain the demand functions of platform *A* and platform *B* with respect to the supply side and the demand side under scenario MN

(6) $$\begin{array}{cc} n_{A}^{I-MN} & =\frac{\alpha \left(\alpha -w_{B}^{MN}+B-t\right)-t\left(A-p_{A}^{MN}\right)}{\alpha^{2}-t^{2}}\\ n_{B}^{I-MN} & =1-\frac{-\alpha A+\alpha w_{A}^{MN}+t\left(\alpha -p_{B}^{MN}+B-t\right)}{\alpha^{2}-t^{2}}\\ n_{A}^{II-MN} & =\frac{\alpha \left(\alpha -p_{B}^{MN}+B-t\right)-t\left(A-w_{A}^{MN}\right)}{\alpha^{2}-t^{2}}\\ n_{B}^{II-MN} & =1-\frac{\alpha \left(p_{A}^{MN}-A\right)+t\left(\alpha -w_{B}^{MN}+B-t\right)}{\alpha^{2}-t^{2}} \end{array}$$

Stage 2.2.2: Under the blockchain strategy, for a type η blockchain technology-sensitive users, a multi-homing demander located at $x_{A}$ on the side *X* (respectively a multi-homing supplier located at $y_{A}$ on the side *Y*) receives utility from joining platforms:

(7) $$\begin{array}{cc} U_{A\&B}^{I-MY} & =\theta \left(A+B\right)+\alpha -p_{A}^{MY}-p_{B}^{MY}-t\\ U_{A\&B}^{II-MY} & =\theta \left(A+B\right)+\alpha -w_{A}^{MY}-w_{B}^{MY}-t \end{array}$$

In such a case, the utility of users only joining platform *A* is given by $U_{A\&B}^{\iota -M\Gamma }-U_{B}^{\iota -M\Gamma }$, (ι∈{I,II},Γ∈{N,Y}). To characterize the size of the market captured by platform *A*, we need to identify the users who is indifferent between joining platform *A* in addition to *B*, and staying with *B* only. This segment of users (${\bar{x}}_{A},{\bar{y}}_{A}$) is characterized by $U_{A\&B}^{\iota -M\Gamma }=U_{B}^{\iota -M\Gamma }$, (ι∈{I,II},Γ∈{N,Y}).

Then we obtain the demand functions of platform *A* and platform *B* with respect to the supply side and the demand side under scenario MY

(8) $$\begin{array}{cc} n_{A}^{I-MY} & =\frac{-\alpha^{2}-tp_{A}^{MY}+A\eta \theta t-A\eta t+At-\alpha B\eta \theta +\alpha B\eta -\alpha B+\alpha w_{B}^{MY}+\alpha t}{t^{2}-\alpha^{2}}\\ n_{B}^{I-MY} & =\frac{-\alpha^{2}-tw_{A}^{MY}+A\eta \theta t-A\eta t+At-\alpha B\eta \theta +\alpha B\eta -\alpha B+\alpha p_{B}^{MY}+\alpha t}{t^{2}-\alpha^{2}}\\ n_{A}^{II-MY} & =\frac{-\alpha^{2}-\alpha A\eta \theta +\alpha A\eta -\alpha A+\alpha w_{A}^{MY}-tp_{B}^{MY}+B\eta \theta t-B\eta t+Bt+\alpha t}{t^{2}-\alpha^{2}}\\ n_{B}^{II-MY} & =\frac{-\alpha^{2}-\alpha A\eta \theta +\alpha A\eta -\alpha A+\alpha p_{A}^{MY}-tw_{B}^{MY}+B\eta \theta t-B\eta t+Bt+\alpha t}{t^{2}-\alpha^{2}} \end{array}$$

Stage 3: In this stage, platforms will reveal the service fees of both the supplier and the demander simultaneously to maximize their profits. In addition, to ensure that all variables are non-negative, we assume that t>α, which is a common assumption in the competition model based on hotelling line. Specifically, the objective functions of the platforms are as follows:

(9) $$\begin{array}{c} Max\;\pi_{i}^{ℸN}=w_{i}^{ℸN}n_{i}^{II-ℸN}+p_{i}^{ℸN}n_{i}^{I-ℸN},ℸ\in \left\{S,M\right\},i\in \left\{A,B\right\} \end{array}$$

Under the blockchain strategy, they need to pay a one-time introduction fee *f*, and thus the objective functions are converted to:

(10) $$\begin{array}{c} Max\;\pi_{i}^{ℸY}=w_{i}^{ℸY}n_{i}^{II-ℸY}+p_{i}^{ℸY}n_{i}^{I-ℸY}-f,ℸ\in \left\{S,M\right\},i\in \left\{A,B\right\} \end{array}$$

## 4. Equilibrium Analysis

To investigate the timing of introducing blockchain technology to sharing economy platforms under different consumer behaviours. We first calculate and analyse equilibrium results for supply and demand bilateral users as single-homing, based on which we extend the analysis to equilibrium results for users as multi-homing. In addition, we have compared and analysed the impact of the timing of the introduction of different blockchain technologies on the pricing and performance levels of sharing economy platforms. The specific solution process is described below. The first step is to deduce the best service fee for the operation strategy selected by the sharing economy platform. To this end, the demand function of the demand side and the supply side derived from the hotelling line is substituted into the profit function. By simultaneously solving the first-order conditions of the profit functions of the two platforms with respect to $w_{i},i\in \left\{A,B\right\}$ and $p_{i},i\in \left\{A,B\right\}$, we can get the function about the service fee. Since we focus on the platform’s blockchain technology introduction strategy in different user markets, we discuss the platform’s equilibrium pricing under different strategies in two different markets with single-homing and multi-homing. Thus, the equilibrium service fees are $w_{i}^{SN},p_{i}^{SN}$ and $w_{i}^{MN},p_{i}^{MN}$ under the strategy of no-introducing blockchain technology; $w_{i}^{SY},p_{i}^{SY}$ and $w_{i}^{MY},p_{i}^{MY}$ under the strategy of introducing blockchain technology.

### 4.1. Equilibrium Analysis in a Single-Homing Case

We first analyze the case of single-homing market, that is, both the demand side and the supply side can only choose a suitable platform for trading services. Furthermore, through a sensitivity analysis of the equilibrium results, we discuss the impact of market factors such as cross-network externalities, the degree of brand differentiation, and the extent to which sensitive consumers perceive the value of the blockchain on the decision making and performance of sharing economy platforms. Finally, we discuss the impact of blockchain introduction decisions on platform pricing and services subject to various degrees of blockchain perception by sensitive consumers.

#### 4.1.1. Equilibrium Outcome in a Single-Homing Market

**Theorem** **1.** *In single-homing markets where the users possess strong brand preferences and be loyal to the platform. Equilibrium service fees when platforms do not introduce the blockchain technology are given by:*

$$\begin{array}{c} p_{A}^{SN}=w_{A}^{SN}=\frac{1}{3}\left(-3\alpha +A-B+3t\right)\;\;;\;\;p_{B}^{SN}=w_{B}^{SN}=\frac{1}{3}\left(-3\alpha -A+B+3t\right) \end{array}$$

*The equilibrium transaction volumes for the supply side and demand side can be obtained as follows:*

$$\begin{array}{c} D_{A}^{I-SN}=D_{A}^{II-SN}=\frac{1}{6}\left(\frac{A-B}{t-\alpha }+3\right)\;\;;\;\;D_{B}^{I-SN}=D_{B}^{II-SN}=\frac{1}{6}\left(\frac{B-A}{t-\alpha }+3\right) \end{array}$$

On the basis of Theorem 1, we can derive the profits of the two platforms in the case of SN as follows:

$$\begin{array}{c} \pi_{A}^{SN}=\pi_{B}^{SN}=\frac{{\left(3t-3\alpha +A-B\right)}^{2}}{9(t-\alpha )} \end{array}$$

To ensure that all variables are nonnegative, we assume that t>α. Furthermore, observe that under this situation, the profits of two asymmetric competitive sharing economic platforms are the same.

**Theorem** **2.** *In single-homing markets where the users possess strong brand preferences and be loyal to the platform. Equilibrium service fees and transaction volumes when platforms introduce the blockchain technology are given by*

$$\begin{array}{c} p_{A}^{SY}=w_{A}^{SY}=\frac{A\eta (\theta -1)+A+B(\eta (1-\theta )-1)+3(t-\alpha )}{3}\;\;;\\ p_{B}^{SY}=w_{B}^{SY}=\frac{B\eta (\theta -1)+B+A(\eta (1-\theta )-1)+3(t-\alpha )}{3} \end{array}$$

*The equilibrium transaction volumes for the supply side and demand side can be obtained as follows:*

$$\begin{array}{c} D_{A}^{I-SY}=D_{A}^{II-SY}=\frac{A\eta (\theta -1)+A+B(\eta (1-\theta )-1)+3(t-\alpha )}{6(t-\alpha )}\;\;;\\ D_{B}^{I-SY}=D_{B}^{II-SY}=\frac{B\eta (\theta -1)+B+A(\eta (1-\theta )-1)+3(t-\alpha )}{6(t-\alpha )} \end{array}$$

On the basis of Theorem 2, we can derive the profits of the two platforms in the case of SY as follows:

$$\begin{array}{cc} \pi_{A}^{SY} & =\frac{{\left(A\eta \left(\theta -1\right)+A\right)}^{2}+{\left(B\eta \left(\theta -1\right)+B\right)}^{2}-6B\left(\eta \left(\theta -1\right)+1\right)\left(t-\alpha \right)-9\left(t-\alpha \right)\left(\alpha +f-t\right)}{9(t-\alpha )}\\ & -\frac{2A(\eta (\theta -1)+1)(3\alpha +B\eta (\theta -1)+B-3t)}{9(t-\alpha )}\\ \pi_{B}^{SY} & =\frac{{\left(A\eta \left(\theta -1\right)+A\right)}^{2}+{\left(B\eta \left(\theta -1\right)+B\right)}^{2}-6B\left(\eta \left(\theta -1\right)+1\right)\left(t-\alpha \right)-9\left(t-\alpha \right)\left(\alpha +f-t\right)}{9(t-\alpha )}\\ & -\frac{2A(\eta (\theta -1)+1)(3t-3\alpha +B\eta (\theta -1)+B)}{9(t-\alpha )} \end{array}$$

Similarly, to ensure that all variables are nonnegative, we assume that t>α. However, under this situation, the profits of two asymmetric competitive sharing economic platforms are no longer the same.

Intuitively, Theorems 1 and 2 show that a unique equilibrium exists, in which supply matches demand, including transaction times and service price. We observe that the platform’s service price and final transaction volume for both the supplier and the demander depend on the utility of the cross-border network, the difference in brand value and the perception of sensitive users’ value. Thus, from Theorem 1 to 2, we have the following:

#### 4.1.2. Sensitivity Results in Terms of Single-Homing Market

**Corollary** **1.** *(a)$\frac{\partial p_{A}^{S\Gamma }}{\partial \alpha }=\frac{\partial w_{A}^{S\Gamma }}{\partial \alpha }=\frac{\partial p_{B}^{S\Gamma }}{\partial \alpha }=\frac{\partial w_{B}^{S\Gamma }}{\partial \alpha }<0,\Gamma \in \left\{N,Y\right\}$; (b)$\frac{\partial D_{A}^{I-S\Gamma }}{\partial \alpha }=\frac{\partial D_{A}^{II-S\Gamma }}{\partial \alpha }>0$ and $\frac{\partial D_{B}^{I-S\Gamma }}{\partial \alpha }=\frac{\partial D_{B}^{II-S\Gamma }}{\partial \alpha }<0,\Gamma \in \left\{N,Y\right\}$.*

Corollary 1 shows that in single-homing markets, the impact of network cross-border effect on service price is independent of the strategy of platforms blockchain technology introduction and the differences in brand value, while the network cross-border effect on transaction times is influenced by brand differences. Specifically, pricing of demand side and supply side of the two differentiated platforms decreases with the increase of network externalities. The amount of platform services with high brand value (Platform *A*) increases with the increase of network externalities. Conversely, the amount of platform services with low brand value (Platform *B*) decreases with the increase of network externalities. The trend in the impact of network externalities on service prices and service volumes is consistent for platform with lower brand value, but the exact opposite is true for platform with lower brand value. In addition, the impact of network externalities on their service prices and service volumes is not subject to blockchain technology.

**Corollary** **2.** *(a)$\frac{\partial p_{A}^{S\Gamma }}{\partial \psi }=\frac{\partial w_{A}^{S\Gamma }}{\partial \psi }>0$ and $\frac{\partial p_{B}^{S\Gamma }}{\partial \psi }=\frac{\partial w_{B}^{S\Gamma }}{\partial \psi }<0,\Gamma \in \left\{N,Y\right\}$; (b) $\frac{\partial D_{A}^{I-S\Gamma }}{\partial \psi }=\frac{\partial D_{A}^{II-S\Gamma }}{\partial \psi }>0$ and $\frac{\partial D_{B}^{I-S\Gamma }}{\partial \psi }=\frac{\partial D_{B}^{II-S\Gamma }}{\partial \psi }<0,\Gamma \in \left\{N,Y\right\}$.*

Corollary 2 shows the influence of brand differentiation on platform transaction times and service price. For platform with high brand value, both service price and transaction times are directly proportional to the degree of platform differentiation, while for platform with low brand value, both service price and transaction times are inversely proportional to the degree of platform differentiation. This means that the greater the degree of brand differentiation, the better the platform with high brand value which is also labeled as “Matthew Effect”. The Matthew Effect is also a widespread market phenomenon in the brand capital sector: the strongest are stronger and the weakest are weaker. This is also one of the reasons to stimulate the platform to pay attention to brand effect.

**Corollary** **3.** *(a) if θ>1, then $\frac{\partial p_{A}^{SY}}{\partial \lambda }=\frac{\partial w_{A}^{SY}}{\partial \lambda }>0$, $\frac{\partial p_{B}^{SY}}{\partial \lambda }=\frac{\partial w_{B}^{SY}}{\partial \lambda }<0$, and $\frac{\partial D_{A}^{I-SY}}{\partial \lambda }=\frac{\partial D_{A}^{II-SY}}{\partial \lambda }>0$, $\frac{\partial D_{B}^{I-SY}}{\partial \lambda }=\frac{\partial D_{B}^{II-SY}}{\partial \lambda }<0$;*

*(b) if 0<θ<1, then $\frac{\partial p_{A}^{SY}}{\partial \lambda }=\frac{\partial w_{A}^{SY}}{\partial \lambda }<0$, $\frac{\partial p_{B}^{SY}}{\partial \lambda }=\frac{\partial w_{B}^{SY}}{\partial \lambda }>0$, and $\frac{\partial D_{A}^{I-SY}}{\partial \lambda }=\frac{\partial D_{A}^{II-SY}}{\partial \lambda }<0$, $\frac{\partial D_{B}^{I-SY}}{\partial \lambda }=\frac{\partial D_{B}^{II-SY}}{\partial \lambda }>0$.*

where λ=ηθ represents sensitive users’ perception of blockchain value. The larger the value of λ, the more sensitive users and the higher the value perception of blockchain.

Thus, from the Corollary 3, we can clearly deduce the following opinions when platform introduce blockchain technology. When the sensitive users’ perceived value of blockchain is at a high level (θ>1), for platforms with higher brand value, the service volume and service price are increasing in the sensitive users’ perceived value coefficient, while for platforms with low brand value, transaction times and service price are decreasing in the sensitive users’ value perception coefficient. However, when the sensitive users’ perceived value of blockchain is at a low level (0<θ<1), the opposite conclusion has been reached. For platforms with higher brand value, the strength of the sensitive users’ perception of blockchain value serves to decrease transaction times and service price, while for platforms with low brand value, the strength of the sensitive users’ perception of blockchain value serves to increase transaction times and service price.

Next, we compare the equilibrium results of the competitive shared economic platforms under two strategies in the single-homing market and describe how the equilibrium strategy responds to the real-time demand changes.

#### 4.1.3. Comparison of Market Factors under Different Strategies in a Single-Homing Market

**Proposition** **1.** *In single-homing markets, under the different strategies of blockchain technology introduction, the following platform service optimal pricing hold:*

*(a)*  *If θ>1, then $p_{B}^{SY}<p_{B}^{SN},w_{B}^{SY}<w_{B}^{SN},p_{A}^{SY}>p_{A}^{SN},w_{A}^{SY}>w_{A}^{SN}$*
*(b)*  *If 0<θ<1, then $p_{B}^{SY}>p_{B}^{SN},w_{B}^{SY}>w_{B}^{SN},p_{A}^{SY}<p_{A}^{SN},w_{A}^{SY}<w_{A}^{SN}$*

As stated in Proposition 1, depending on sensitive users’ different perceptions of blockchain value, the adoption of blockchain technology will lead to different impacts on the pricing of platforms with asymmetric brand value. When sensitive user’s perception of blockchain value is high (θ>1), the strategy of introducing blockchain technology will make the platform with high (respectively, low) brand value increase (respectively, decrease) its pricing for both supply side and demand side. Furthermore, if sensitive users’ perception of blockchain value is low (0<θ<1), adopting the blockchain technology will result in a higher (respectively, low) price for the platform with low (respectively, high) brand value under equilibrium.

**Proposition** **2.** *In single-homing markets, under the different strategies of blockchain technology introduction, the following platform optimal transaction times hold:*

*(a)*  *If θ>1, then $D_{A}^{I-SY}>D_{A}^{I-SN},D_{A}^{II-SY}>D_{A}^{II-SN},D_{B}^{I-SY}<D_{B}^{I-SN},D_{B}^{II-SY}<D_{B}^{II-SN}$*
*(b)*  *If 0<θ<1, then $D_{A}^{I-SY}<D_{A}^{I-SN},D_{A}^{II-SY}<D_{A}^{II-SN},D_{B}^{I-SY}>D_{B}^{I-SN},D_{B}^{II-SY}>D_{B}^{II-SN}$*

Proposition 2 implies that the strategy of introducing blockchain technology will lead the same change trend of transaction times on both supply and demand sides of each platform. Specifically, if sensitive users’ perception of blockchain value is at a high (respectively, low) level, the strategy of introducing blockchain technology will induce different effects on the competition platforms: the platform with high brand value will gain a higher (respectively, lower) transaction times, while the platform with low brand value will gain a lower (respectively, higher) transaction times.

### 4.2. Equilibrium Analysis in a Multi-Homing Case

In this section, we analyze multi-homing market based on the model of singling-homing market, that is, there are some suppliers and demanders who choose to trade on two platforms. We prove that there is a unique equilibrium even if there is the multi-homing market. Further, we address how market factors such as cross-network externalities, the degree of brand differentiation, and the degree to which sensitive consumers perceive the value of blockchain affect the decision making and performance of sharing economy platforms under multi-homing market condition in a similar and different way compared to single-homing market. Finally, we discuss the impact of blockchain introduction decisions on platform pricing and services and find that the introduction of blockchain technology is more constrained by the degree of brand differentiation in multi-homing markets compared to single-homing markets.

#### 4.2.1. Equilibrium Outcome in a Multi-Homing Market

**Theorem** **3.** *In multi-homing market conditions, users choose to transact services on both platforms simultaneously. Equilibrium service fees and transaction times under the case of MN are given by*

$$\begin{array}{cc} p_{A}^{MN} & =w_{A}^{MN}=\alpha +A+t+\frac{t(A+B+4t)}{2(\alpha -2t)}-\frac{t(A-B)}{2(\alpha +2t)}\;\;;\;\;D_{A}^{I-MN}=D_{A}^{II-MN}=\frac{t\left(-\alpha^{3}+2At^{2}-\alpha^{2}\left(A+t\right)+\alpha t\left(2t-B\right)\right)}{\alpha^{4}+4t^{4}-5\alpha^{2}t^{2}}\\ p_{B}^{MN} & =w_{B}^{MN}=\alpha +B+t+\frac{t(A+B+4t)}{2(\alpha -2t)}+\frac{t(A-B)}{2(\alpha +2t)}\;\;;\;\;D_{B}^{I-MN}=D_{B}^{II-MN}=\frac{t\left(-\alpha^{3}+\alpha t\left(2t-A\right)+2Bt^{2}-\alpha^{2}\left(B+t\right)\right)}{\alpha^{4}+4t^{4}-5\alpha^{2}t^{2}} \end{array}$$

On the basis of Theorem 3, we can derive the profits of the two platforms in the case of MN as follows:

$$\begin{array}{c} \pi_{A}^{MN}=\frac{2t{\left(\alpha t\left(B-2t\right)+\alpha^{3}-2At^{2}+\alpha^{2}\left(A+t\right)\right)}^{2}}{\left(t^{2}-\alpha^{2}\right){\left(\alpha^{2}-4t^{2}\right)}^{2}}\\ \pi_{B}^{MN}=\frac{2t{\left(\alpha^{3}+\alpha t\left(A-2t\right)-2Bt^{2}+\alpha^{2}\left(B+t\right)\right)}^{2}}{\left(t^{2}-\alpha^{2}\right){\left(\alpha^{2}-4t^{2}\right)}^{2}} \end{array}$$

To ensure that all variables are nonnegative and the multi-homing market exists, we assume that t>α and $A>B>\frac{-\alpha^{2}+2t^{2}-\alpha t}{t}$. Note that under multi-homing market, the profits of two asymmetric competitive sharing economic platforms are no longer the same, whereas under single-homing market, the profits of two asymmetric competitive sharing economic platforms are the same.

**Theorem** **4.** *In single-homing markets where the users pay the service fees of both platform. Equilibrium service fees and trading volumes under the case of MY are given by*

$$\begin{array}{cc} p_{A}^{MY} & =w_{A}^{MY}=\frac{A\left(\eta \left(\theta -1\right)+1\right)\left(2t^{2}-\alpha^{2}\right)-\alpha \left(\alpha^{2}+Bt\left(\eta \left(\theta -1\right)+1\right)-2t^{2}+\alpha t\right)}{4t^{2}-\alpha^{2}}\\ p_{B}^{MY} & =w_{B}^{MY}=\frac{B\left(\eta \left(\theta -1\right)+1\right)\left(2t^{2}-\alpha^{2}\right)-\alpha \left(\alpha^{2}+At\left(\eta \left(\theta -1\right)+1\right)-2t^{2}+\alpha t\right)}{4t^{2}-\alpha^{2}}\\ D_{A}^{I-MY} & =D_{A}^{II-MY}=\frac{t\left(A\left(\eta \left(\theta -1\right)+1\right)\left(2t^{2}-\alpha^{2}\right)-\alpha \left(\alpha^{2}+Bt\left(\eta \left(\theta -1\right)+1\right)-2t^{2}+\alpha t\right)\right)}{\alpha^{4}+4t^{4}-5\alpha^{2}t^{2}}\\ D_{B}^{I-MY} & =D_{B}^{II-MY}=\frac{t\left(B\left(\eta \left(\theta -1\right)+1\right)\left(2t^{2}-\alpha^{2}\right)-\alpha \left(\alpha^{2}+At\left(\eta \left(\theta -1\right)+1\right)-2t^{2}+\alpha t\right)\right)}{\alpha^{4}+4t^{4}-5\alpha^{2}t^{2}} \end{array}$$

On the basis of Theorem 4, we can derive the profits of the two platforms in the case of MY as follows:

$$\begin{array}{c} \pi_{A}^{MY}=\frac{\frac{2t{\left(\alpha \left(\alpha^{2}+Bt\left(\eta \left(\theta -1\right)+1\right)-2t^{2}+\alpha t\right)-A\left(\eta \left(\theta -1\right)+1\right)\left(2t^{2}-\alpha^{2}\right)\right)}^{2}}{{\left(\alpha^{2}-4t^{2}\right)}^{2}}+f\left(\alpha^{2}-t^{2}\right)}{t^{2}-\alpha^{2}}\\ \pi_{B}^{MY}=\frac{\frac{2t{\left(\alpha \left(\alpha^{2}+At\left(\eta \left(\theta -1\right)+1\right)-2t^{2}+\alpha t\right)-B\left(\eta \left(\theta -1\right)+1\right)\left(2t^{2}-\alpha^{2}\right)\right)}^{2}}{{\left(\alpha^{2}-4t^{2}\right)}^{2}}+f\left(\alpha^{2}-t^{2}\right)}{t^{2}-\alpha^{2}} \end{array}$$

To ensure that all variables are nonnegative and the multi-homing market exists, we assume that t>α and $A>B>\frac{-\alpha^{2}+2t^{2}-\alpha t}{t\theta }$. Note that the introduction of blockchain in multi-homing market conditions results in the profits of the two asymmetric platforms no longer remaining equal, which also occurs in a single-homing market.

#### 4.2.2. Sensitivity Results in Terms of Multi-Homing Market

**Corollary** **4.** *(a)$\frac{\partial p_{A}^{M\Gamma }}{\partial \psi }=\frac{\partial w_{A}^{M\Gamma }}{\partial \psi }>0$ and $\frac{\partial p_{B}^{M\Gamma }}{\partial \psi }=\frac{\partial w_{B}^{M\Gamma }}{\partial \psi }<0,\Gamma \in \left\{N,Y\right\}$; (b) $\frac{\partial D_{A}^{I-M\Gamma }}{\partial \psi }=\frac{\partial D_{A}^{II-M\Gamma }}{\partial \psi }>0$ and $\frac{\partial D_{B}^{I-M\Gamma }}{\partial \psi }=\frac{\partial D_{B}^{II-M\Gamma }}{\partial \psi }<0,\Gamma \in \left\{N,Y\right\}$.*

Corollary 4 (a) points out that how brand value difference affects platform pricing in a multi-homing market. Corollary 4 (b) presents the influence of brand value difference on platform transaction volumes in a multi-homing market. More precisely, as the brand differentiation increases, platform with higher brand value sets higher service fees and gets higher transaction times, while the situation of platform pricing and transaction times with inferior brand value is completely opposite. In addition, as in a single-homing market, brand differentiation has the same trend of impact on platform service prices and service volumes under multi-homing market.

**Corollary** **5.** *(a) if $\theta >1,\psi <\frac{2t^{2}-\alpha^{2}}{\alpha t}$, then $\frac{\partial p_{A}^{MY}}{\partial \lambda }=\frac{\partial w_{A}^{MY}}{\partial \lambda }<0$ and $\frac{\partial p_{B}^{MY}}{\partial \lambda }=\frac{\partial w_{B}^{MY}}{\partial \lambda }>0$; if $\theta >1,\psi >\frac{2t^{2}-\alpha^{2}}{\alpha t}$, then $\frac{\partial p_{A}^{MY}}{\partial \lambda }=\frac{\partial w_{A}^{MY}}{\partial \lambda }>0$ and $\frac{\partial p_{B}^{MY}}{\partial \lambda }=\frac{\partial w_{B}^{MY}}{\partial \lambda }<0$; if $0<\theta <1,\psi <\frac{2t^{2}-\alpha^{2}}{\alpha t}$, then $\frac{\partial p_{A}^{MY}}{\partial \lambda }=\frac{\partial w_{A}^{MY}}{\partial \lambda }>0$, $\frac{\partial p_{B}^{MY}}{\partial \lambda }=\frac{\partial w_{B}^{MY}}{\partial \lambda }<0$; if $0<\theta <1,\psi >\frac{2t^{2}-\alpha^{2}}{\alpha t}$, then $\frac{\partial p_{A}^{MY}}{\partial \lambda }=\frac{\partial w_{A}^{MY}}{\partial \lambda }<0$, $\frac{\partial p_{B}^{MY}}{\partial \lambda }=\frac{\partial w_{B}^{MY}}{\partial \lambda }>0$; (b) if θ>1, $\frac{\partial D_{A}^{I-MY}}{\partial \lambda }=\frac{\partial D_{A}^{II-MY}}{\partial \lambda }>0$, $\frac{\partial D_{B}^{I-MY}}{\partial \lambda }=\frac{\partial D_{B}^{II-MY}}{\partial \lambda }<0$; if 0<θ<1, $\frac{\partial D_{A}^{I-MY}}{\partial \lambda }=\frac{\partial D_{A}^{II-MY}}{\partial \lambda }<0$, $\frac{\partial D_{B}^{I-MY}}{\partial \lambda }=\frac{\partial D_{B}^{II-MY}}{\partial \lambda }>0$.*

Corollary 5 (a) provides a sufficient condition for platforms pricing under the introduction of blockchain technology in the multi-homing market. Specifically, we first consider users who have a high perception of blockchain value. When the brand difference is large, the pricing of the platform with larger (respectively, lower) brand value increases (respectively, decreases) with the proportion of blockchain sensitive users. Otherwise, When the brand difference is small, the pricing of the platform with lower (respectively, higher) brand value decreases (respectively, increases) with the proportion of blockchain sensitive users. Then we discuss users with low perception of blockchain value. A smaller (respectively, larger) brand difference will contribute to raising (respectively, reducing) the price of platform with higher brand value.

Corollary 5 (b) shows the effect of sensitive users proportion on platform transaction times under the introduction of blockchain technology in the multi-homing market. As the number of sensitive users with high perception of blockchain value is large, more users will choose to trade on the platform with higher brand value. Respectively, with a higher number of sensitive users of low blockchain value perception, more users tend to choose to transact on platforms with lower brand value.

#### 4.2.3. Comparison of Market Factors under Different Strategies in a Multi-Homing Market

**Proposition** **3.** *In a multi-homing markets, when the sensitive users of the blockchain are within a certain range, e.g., $\eta \in (\min\left\{0,\eta_{1}\right\},\max\left\{\eta_{1},1\right\})$, the platform service optimal pricing under the different strategies of blockchain technology introduction is as follows:*

*(a)*  *If $\theta >1,1<\psi <\frac{2t^{2}-\alpha^{2}}{\alpha t}$, then $p_{B}^{MY}<p_{B}^{MN},w_{B}^{MY}<w_{B}^{MN},p_{A}^{MY}>p_{A}^{MN},w_{A}^{MY}>w_{A}^{MN}$*
*(b)*  *If $0<\theta <1,\psi >\frac{2t^{2}-\alpha^{2}}{\alpha t}$, then $p_{B}^{MY}>p_{B}^{MN},w_{B}^{MY}>w_{B}^{MN},p_{A}^{MY}<p_{A}^{MN},w_{A}^{MY}<w_{A}^{MN}$*

By Proposition 3, we find that the pricing comparison between the blockchain strategies of two platforms in a multi-homing market depends on the proportion of sensitive users and their perception of blockchain value, and the differentiation of platforms. Specifically, Proposition 3 presents a case in which the price under blockchain strategy is higher (respectively, lower) than under no-blockchain strategy for the platform with high (respectively, low) brand value. This case holds when the difference of brand value is small and sensitive users have high perception of blockchain value. In fact, in the case of platform with high brand value, the optimal price set under the blockchain strategy is relatively high, so as to extract surplus from a relatively large part of users with high independent valuation.

Now we are going to show that transaction times comparison between the blockchain strategies of two platforms in a multi-homing market.

**Proposition** **4.** *In a multi-homing market, when the sensitive users of the blockchain are within a certain range, e.g., $\eta \in (\min\left\{0,\eta_{1}\right\},\max\left\{\eta_{1},1\right\})$, the platform service optimal transaction times under the different strategies of blockchain technology introduction is as follows:*

*(a)*  *If $\theta >1,1<\psi <\frac{2t^{2}-\alpha^{2}}{\alpha t}$, then $D_{A}^{I-MY}<D_{A}^{I-MN},D_{A}^{II-MY}<D_{A}^{II-MN},D_{B}^{I-MY}>D_{B}^{I-MN},D_{B}^{II-MY}>D_{B}^{II-MN}$*
*(b)*  *If $0<\theta <1,\psi >\frac{2t^{2}-\alpha^{2}}{\alpha t}$, then $D_{A}^{I-MY}>D_{A}^{I-MN},D_{A}^{II-MY}>D_{A}^{II-MN},D_{B}^{I-MY}<D_{B}^{I-MN},D_{B}^{II-MY}<D_{B}^{II-MN}$*

Proposition 4 suggests that when brand differentiation is quite small, the number of transactions under blockchain strategy is lower than under no-blockchain strategy for platforms with high brand value. However, in the case of platform with low brand value, the introduction of blockchain technology results in a higher number of transactions for itself. In contrast, platform with higher brand value will receive higher transaction volumes under a blockchain strategy when the brand differentiation is greater. At this point, platform with low brand value will result in lower transaction volume for itself by introducing blockchain technology.

## 5. Strategy Analysis

In the above discussions, we have obtained comparative results of blockchain strategies for asymmetric platforms under different market conditions. In this section, we continue to compare the applicable conditions of optimal strategy and equilibrium strategy for two differentiated platforms in terms of a single-homing market and multi-homing market.

### 5.1. Optimal Strategy under a Single-Homing Market

**Proposition** **5.** *The optimal strategy for platform A in a single-homing market is as follows:*

- *Choose introducing the blockchain technology if and only if $f<\phi_{A}^{S}$ and θ>1.*
- *Choose not introducing the blockchain technology if $f>\phi_{A}^{S}$ or 0<θ<1.*

*Moreover, define $\phi_{A}^{S}$ as the root of $\pi_{A}^{SN}=\pi_{A}^{SY}$.*

Note that $\phi_{A}^{S}=\frac{\eta (\theta -1)(A-B)(A(\eta (\theta -1)+2)+B(\eta (-\theta )+\eta -2)+6(t-\alpha ))}{9(t-\alpha )}$.

From Proposition 5, we find that when the introduction cost of blockchain exceeds a threshold value, $\phi_{A}^{S}$, the platform with high brand value is more inclined to the conventional operation strategy, that is, it does not introduce blockchain technology. Thus, the platform will choose the “*N*“ scheme. When the introduction cost of blockchain is relativity small, $f<\phi_{A}^{S}$, the platform will adopt the “*Y*“ scheme to attract more sensitive users which have a high perception of blockchain value, θ>1. In particular, only when sensitive users have a high perception of blockchain value does platform with higher brand value introduce blockchain technology.

**Figure 4:**
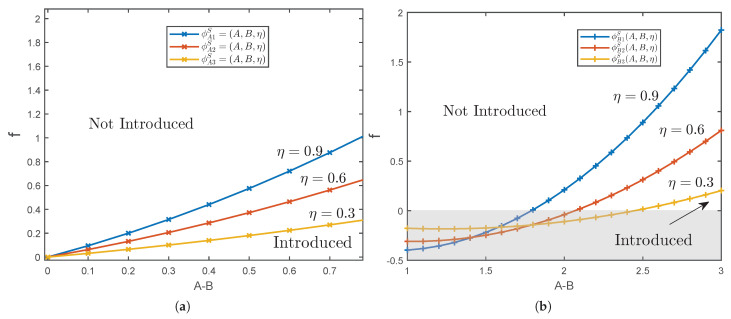
Optima Strategy in Single-homing Markets. (**a**) Platform A’s Optimal Strategy. (**b**) Platform B’s Optimal Strategy.

For a clearer demonstration of the timing of platform blockchain technology introduction, the optimal strategy for platform *A* in a single-homing market under various conditions is shown in Figure 4a. The values of the parameters are set as θ=2.5, t=1.5, α=0.5. Based on this, we consider the timing of platform A blockchain technology introduction under different introduction costs and platform differentiation when sensitive users account for η=0.3, η=0.6 and η=0.9 separately. Combined with Proposition 5, we can find that the maximum cost threshold for platforms to implement a blockchain strategy gradually rises as platform differentiation expands. Furthermore, as the sensitive users increases, the cost threshold for implementing blockchain strategies on the platform gradually is enhanced.

The intuition for platform strategy selection is as follow: only when sensitive users overestimate the value of blockchain will platform *A* introduce blockchain technology. When the sensitive users underestimate the value of blockchain, the brand advantage of platform *A* will be weakened. In addition, the technical advantage brought by blockchain is far lower than the loss of brand value. Since users are single-homing users, the loss of users does great harm to platforms. Therefore, in this case, platform *A* tends not to introduce blockchain technology. Conversely, platform *A* introduces blockchain when sensitive users in the market are the highly valued type, not only does it gain a technological advantage, but it also expands its intrinsic brand value. Consequently, it is always advisable for platform *A* to introduce blockchain technology in such market circumstances as long as the cost of introducing blockchain technology is within an acceptable range.

**Proposition** **6.** *The optimal strategy for platform B in a single-homing market is as follows:*

- *when θ>1 exists, introduce the blockchain technology if and only if $f<\phi_{B}^{S},\alpha <t<t_{1},\eta >\eta_{1}$.*
- *when 0<θ<1 exists, introduce the blockchain technology if and only if $f<\phi_{B}^{S},t>t_{1},0<\eta <\eta_{1}$.*

Note that $\phi_{B}^{S}=\frac{\eta (\theta -1)(A-B)(6\alpha +A(\eta (\theta -1)+2)+B(\eta (-\theta )+\eta -2)-6t)}{9(t-\alpha )}$.

Proposition 6 informs that the platform with low brand value in a single-homing market may benefit from the blockchain scheme. As mentioned before, whether the platform *B* implements blockchain strategy is related to technology introduction cost, user behavior characteristics (perceived value of blockchain and the scale of sensitive users) and distance matching cost. We notice that low brand value platform has lower blockchain value perception requirement for sensitive users than high brand value platform, that is, platform B has the alternative to introduce blockchain technology at an appropriate time to generate more revenue, no matter whether the value perception of sensitive users is high or low.

Under the condition where sensitive users have high perception of blockchain value, platform with low brand value have the potential to introduce blockchain, which requires that (1) The introduction cost is relatively low (i.e., $f<\phi_{B}^{S}$), (2) sensitive users are relatively large in scale (i.e., $\eta >\eta_{1}$), and (3) distance matching cost is relatively low (i.e., $\alpha <t<t_{1}$), that is, the penalty cost for users to renege is high. Moreover, when sensitive users have low perception of blockchain value, the platform still has the potential to introduce blockchain technology. This case holds that (1) The introduction cost is relatively low (i.e., $f<\phi_{B}^{S}$); however, (2) sensitive users are relatively small in scale (i.e., $0<\eta <\eta_{1}$), and (3) distance matching cost is relatively high (i.e., $t>t_{1}$).

The rationale is that for platform *B* with weak brand value, when the proportion of sensitive users is relatively high and the perception of blockchain value is low, platform *B* will choose to introduce blockchain technology at an acceptable cost, if the distance matching cost is high. Although the sensitive users underestimate the value of blockchain in this case, the distance matching cost is high, that is, the user’s regret cost is high. Therefore, even though most users are not inclined to choose the platform with blockchain technology, the regret cost is too high for them, so this group of users will not easily change the platform, so the platform *B* can still get higher profits, because the advantages from technology are far higher than the losses from brand value. Nonetheless, Platform *B* may potentially choose to introduce blockchain technology at an appropriate cost when sensitive users are a relatively low percentage of the user base and the perception of the blockchain’s value is relatively high. Specifically, for potential sensitive users with a higher perception of blockchain value, if platform *B* introduces blockchain technology, sensitive users of platform *A* will choose platform *B* under a lower distance cost, so that platform *B* could attract more sensitive users and thus acquire both technology and brand advantages. Hence, in this case, platform *B* is more inclined to introduce blockchain technology.

To show the the timing of platform *B* blockchain technology introduction more intuitively, we assume that the figure of optimal strategy for platform *B* in a single-homing market when θ=2.5, t=1.5, α=0.5, considering different sensitive user scales, such as η=0.3, η=0.6 and η=0.9. From Figure 4b, we find that platform *B* has high requirements for brand differentiation. Only when it is at a great disadvantage in brand, will the platform B try to introduce blockchain technology to improve the status quo of brand disadvantage.

### 5.2. Optimal Strategy under Multi-Homing Market

**Proposition** **7.** *The optimal strategy for platform A in a multi-homing market is as follows:*

- *Choose introducing the blockchain technology if and only if $f<\phi_{A}^{M}$ and θ>1.*
- *Choose not introducing the blockchain technology if $f<\phi_{A}^{M}$ and 0<θ<1.*

Note that $\phi_{A}^{M}=\frac{2t\left({\left(\alpha \left(\alpha^{2}+Bt\left(\eta \left(\theta -1\right)+1\right)-2t^{2}+\alpha t\right)-A\left(\eta \left(\theta -1\right)+1\right)\left(2t^{2}-\alpha^{2}\right)\right)}^{2}-{\left(\alpha^{3}-2At^{2}+\alpha^{2}\left(A+t\right)+\alpha t\left(B-2t\right)\right)}^{2}\right)}{\left(t^{2}-\alpha^{2}\right){\left(\alpha^{2}-4t^{2}\right)}^{2}}$.

Proposition 7 reveals the timing for platform with higher brand value to introduce blockchain when there are multi-homing users in the market. As with the single-homing market, for platform *A* which has high brand value, it will only consider introducing blockchain technology within the appropriate cost threshold level as long as blockchain sensitive users are overvalued. Once sensitive users are undervalued, platform *A* will not introduce blockchain technology even at a low cost. Because the loss of brand utility suffered by platform *A* is far greater than the technical advantage brought by introducing blockchain, it is the best choice for platform *A* not to introduce blockchain on this occasion.

**Figure 5:**
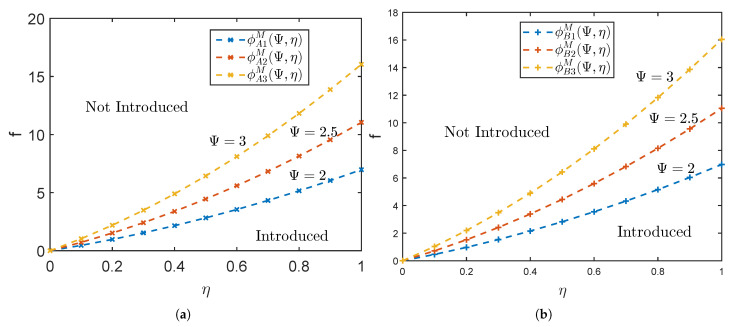
Optimal Strategy in Multi-homing Markets. (**a**) Platform A’s Optimal Strategy. (**b**) Platform B’s Optimal Strategy.

Figure 5a shows the timing of platform *A*’s optimal strategy under multi-homing market. Specifically, we still use the above parameter setting (e.g., θ=2.5, t=1.5, α=0.5) to describe the effect of platform differentiation and introduction cost on the timing of introducing blockchain. Meanwhile, we consider the influence of platform difference rate of 3, 2.5 and 2 on platform blockchain strategy. As Figure 5a shown, the greater the platform differentiation, the higher the threshold of acceptable introduction cost of the platform with higher brand value which will expand the scope of introducing blockchain technology. Accordingly, the acceptable introduction cost of the platform is proportional to the ratio of sensitive users.

**Proposition** **8.** *The optimal strategy for platform B in a multi-homing market is as follows:*

- *when θ>1 exists, introduce the blockchain technology if and only if $f<\phi_{B}^{M}$ and $1<\psi <\frac{2t^{2}-\alpha^{2}}{\alpha t}$.*
- *when 0<θ<1 exists, introduce the blockchain technology if and only if $f<\phi_{B}^{M}$ and $\frac{2t^{2}-\alpha^{2}}{\alpha t}<\psi$.*

Note that $\phi_{B}^{M}=\frac{2t\left({\left(\alpha \left(\alpha^{2}+At\left(\eta \left(\theta -1\right)+1\right)-2t^{2}+\alpha t\right)-B\left(\eta \left(\theta -1\right)+1\right)\left(2t^{2}-\alpha^{2}\right)\right)}^{2}-{\left(\alpha^{3}+\alpha t\left(A-2t\right)-2Bt^{2}+\alpha^{2}\left(B+t\right)\right)}^{2}\right)}{\left(t^{2}-\alpha^{2}\right){\left(\alpha^{2}-4t^{2}\right)}^{2}}$.

Proposition 8 reveals the timing for platform with lower brand value to introduce blockchain when there are multi-homing users in the market. Compared with the single-homing market, the blockchain strategy of the platform which has lower brand value in the multi-homing market is not only limited by the introduction cost, but also focused on the brand value differentiation. It is intuitive that the platform will consider the blockchain strategy when the introduction cost is lower than the threshold (e.g., $f<\phi_{B}^{M}$). However, as sensitive users in the market are highly valued, platform B only adopts blockchain strategy when brand differentiation is small (i.e., $1<\psi <\frac{2t^{2}-\alpha^{2}}{\alpha t}$), while the sensitive users in the market are low-valued, platform B chooses blockchain strategy only when the brand differentiation is large (i.e., $\frac{2t^{2}-\alpha^{2}}{\alpha t}<\psi$).

Figure 5b shows the timing of platform *B*’s optimal strategy under multi-homing market. The parameters are set as in Proposition 7. As with platform *A*, the greater the brand differentiation, the greater the threshold of acceptable introduction cost of platform *B*, and thus the broader the platform blockchain strategy is.

### 5.3. Equilibrium Strategy under Different Market Conditions

**Proposition** **9.** *The equilibrium strategy for platforms is as follows:*

- *In a single-homing market, both platforms introduce the blockchain technology if and only if $\theta >1,t>t_{1},\eta >\eta_{1},f<min\left\{\phi_{A}^{S},\phi_{B}^{S}\right\}$.*
- *In a multi-homing market, both platforms introduce the blockchain technology if and only if $\theta >1,\frac{2t^{2}-\alpha^{2}}{\alpha t}<\psi ,f<min\left\{\phi_{A}^{M},\phi_{B}^{M}\right\}$.*

Proposition 9 indicates that whether in a single-homing market or a multi-homing market, there is a strategic equilibrium. Intuitively, both markets require high valuation of sensitive users (i.e., θ>1) and relatively low introduction cost (i.e., $f<min\{\phi_{A}^{S},\phi_{B}^{S}\}$, $f<min\{\phi_{A}^{M},\phi_{B}^{M}\}$). Conversely, in a single-homing market, platforms are more concerned about the distance cost of users and the scale of sensitive users. That is, in terms of user characteristics, the two asymmetric sharing economic platforms will realize the blockchain strategy equilibrium in the single-homing market, when there are many sensitive users in the market and the regret cost is high enough that they will not easily switch platforms. The rationale is that introducing blockchain technology not only does not lead to a large number of users losing, but also enhances sensitive users’ perception of the platform’ s value and improves its profitability. In a multi-homing market, if the asymmetric platform achieves market equilibrium, it is necessary to ensure significant brand differentiation.

**Figure 6:**
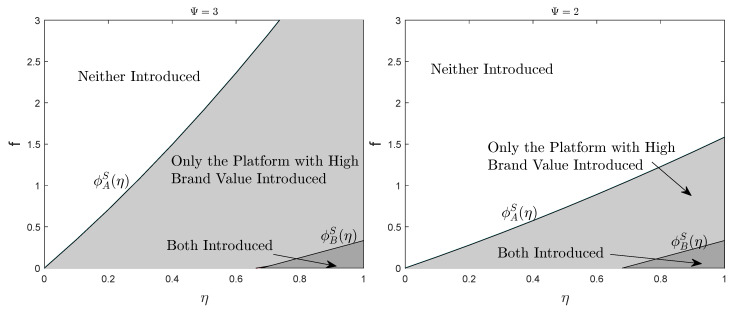
Equilibrium Strategy in Single-homing Markets.

**Figure 7:**
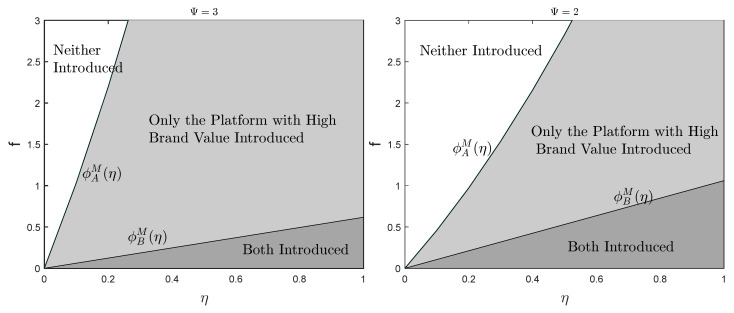
Equilibrium Strategy in Multi-homing Markets.

To provide a more intuitive insight into the impact of various factors on the realization of a balanced market, Figure 6 and Figure 7 complement the results of our analysis. The parameters are set as above. Comparing Figure 6 and Figure 7, it is obviously discovered that the expansion of platform differentiation in a single-homing market has less impact on the formation of blockchain equilibrium than in a multi-homing market. Moreover, the higher the brand value, the higher the threshold of the introduction cost which the platform accepts. Through analysis and comparison, the requirement of single-homing market on the scale of sensitive users is higher than that of multi-homing market. Only when the proportion of sensitive users is higher than the threshold can the blockchain equilibrium situation be formed, while the restriction on the proportion of sensitive users in a multi-homing market is considerably looser.

## 6. Conclusions

In this paper, we analyse the timing of asymmetric sharing economy platforms introducing blockchain technology while considering user characteristics. First, we calculate the optimal pricing and profits of asymmetric platforms under different blockchain strategies in a single-homing market, followed by comparing and analysing the specific timing of the introduction of blockchain technology for asymmetric platforms. We also consider the multi-homing market, comparatively analyse the optimal profit and pricing of asymmetric platforms under different blockchain strategies, and extract the timing of the introduction of blockchain technology for asymmetric platforms in the multi-homing market. Specific conclusions are presented below. (1) Regarding user characteristics, we find that asymmetric platforms have incentives to introduce blockchain technology when sensitive users have a high perception of blockchain value. Nevertheless, sharing economy platform with higher brand value exits the blockchain market when sensitive users’ perception of blockchain value is low, while platform with lower brand value enter the blockchain market only when sensitive users are relatively low. This conclusion applies to both single-homing and multi-homing markets. (2) Concerning asymmetric platforms, we conclude that the degree of differentiation has a greater impact on the blockchain strategy of platform which has low brand value under the condition of multi-homing market. Under market conditions where sensitive users’ value perceptions are high, the profitability of asymmetric platform with low differentiation can be enhanced by the introduction of blockchain technology, while under market conditions where sensitive users’ value perceptions are low, blockchain technology can be introduced into asymmetric platform with high differentiation to improve profitability. (3) As for the introduction cost of blockchain technology, we draw the conclusion that the introduction cost threshold is higher for asymmetric platforms in a multi-homing market than in a single-homing market, as well as for sharing economy platforms with high brand value than those with low brand value. (4) Based on market conditions, we discover that asymmetric platforms are better able to implement blockchain strategy under multi-homing market conditions.

## Figures and Tables

**Table 1:** Summary of Main References.

Articles	Sharing Economy		Blockchain Technology		Multi-Homing	
	Privacy Concerns	Pricing	Trust Level	Application	Single Side	Both Sides
Armstrong and Wright [44]					✓	
Lutz et al. [9]	✓					
Zarifis et al. [3]	✓	✓				
Lumineau et al. [30]	✓		✓	✓		
Wang et al. [29]			✓	✓		
Li and Zhu [46]					✓	✓
Present study	✓	✓	✓	✓		✓

## Data Availability

Not applicable.
